# Insights from losing the autism diagnosis: Autism spectrum disorder as a biological entity

**DOI:** 10.3389/fpsyt.2022.972612

**Published:** 2022-08-16

**Authors:** Inge-Marie Eigsti, Deborah A. Fein

**Affiliations:** ^1^Psychological Sciences, University of Connecticut, Storrs, CT, United States; ^2^Pediatrics, University of Connecticut, Farmington, CT, United States

**Keywords:** loss of autism diagnosis (LAD), developmental trajectories and cascades, response to intervention, outcomes, autism spectrum disorder

## Introduction

There is an explosion of interest in the question of whether autism spectrum disorder (ASD) is a coherent entity, and further, whether this entity maps onto some biological substrate. We propose that much can be learned by studying symptom remission in ASD.

### Defining a syndrome

*Disorders* are defined by the characteristics of *impaired functioning, distress*, and *atypicality*. These characteristics are polythetic; one can have atypicality and impairment, but not distress (e.g., in personality disorders). Further, syndromes (which may not meet criteria for a disorder) are defined by the fact that symptoms co-occur more than would be expected by chance and have presumed common etiologies. For example, many neurologists and neuropsychologists believe firmly in the reality of the Gerstmann syndrome (1, 2), a cluster of three to five symptoms associated with angular gyrus lesions, but Arthur Benton was a skeptic (3). Similarly, while some eminent neurologists believe a specific behavioral syndrome occurs interictally with temporal lobe epilepsy (4), others [e.g., (5)] propose a “simple” elevation of non-specific psychopathology, rather than this personality profile. Thus, even when syndromes have a clear anatomical substrate, and have been studied for decades, the presence of a resulting clinical syndrome can remain controversial. The diagnostic challenges are heightened when one starts with a clinical syndrome and attempts to uncover a biological physiology or etiology, as with autism.

### Categories vs. dimensions

Disorders are generally defined as categorical entities, in which each category member shares the characteristics of that category. The DSM generally has a categorical structure, with the caveat that DSM-5 diagnoses also have specifiers that allow category members to differ on important dimensions such as language impairment and degree of support needed. In the DSM approach, diagnoses consist of complex clusters of symptoms; these complex clusters are difficult to connect to underlying physiology and neurobiology. Rapin (6) eloquently described the challenges associated with mapping a syndrome defined at one level of description (e.g., behavior) onto characteristics at other levels [pathophysiology, etiology (such as, genetics)]; she held that given our current state of knowledge, such mapping was impossible. NIH's Research Domain Criteria [RDoC; (7)] offer an alternative structure, which provides a strategy for discovering lawful relationships linking basic biological processes to behaviors. By decoupling the symptom clusters, RDoC promotes treating each symptom as a continuum and linking individual differences in those symptoms to causal mechanisms.

This continuum approach holds great appeal, in part because it maps well onto our intuitions about many symptoms; there does not appear to be a qualitative difference between momentary anxiety experienced under threat, vs. the daily anxiety experienced in an anxiety disorder. The continuum approach also facilitates access to clinical services and financial supports for those individuals who do not meet full diagnostic criteria under the categorical medical model but might still benefit from services. Another strength of the continuum approach is that its advocates promote a focus on the societal structures that serve to impede or promote autonomy, health, and success, rather than individual-level symptoms (8).

However, the continuum approach masks some important practical advantages of the categorical medical model. Most important, in our view, is the notion of impairment and distress; while everyone experiences feelings of anxiety on occasion, those individuals with anxiety disorders are so affected by their symptoms that they struggle to function; their anxiety prevents them from performing everyday life tasks, impacts their social relationships, and prevents them from performing as successfully as they could in academic and vocational domains. Treating a condition as a set of dimensions makes it more difficult to allocate scarce treatment resources; in contrast, the DSM model helps to identify those who most require treatment or support in order to function. While the precise threshold for treatment is arbitrary, its location can be data-driven, based on long-term outcomes and experiences. Based on our clinical experience, we also fear that, in jettisoning the medical model, we risk ignoring important variance in cognitive and linguistic barriers, which will lead to neglect or harm to some individuals.

### Is autism a syndrome?

Doubts about the coherence of autism as a behavioral syndrome are legion, despite the fact that most research still generally follows the case-control method, where cases of autism are examined as a group. Indeed, the 2014 special issue of *Autism the International Journal of Research and Practice* was devoted to discussions of this question [e.g., (9, 10)]. Waterhouse (11–14) has argued that autism must be “taken apart” in order to map clinical features onto possible etiologies. Waterhouse and Gillberg (12) suggest that very narrowly defined subgroups, both at the phenotypic and biological levels, will increase the probability of being able to link the two domains. In addition, Waterhouse (11) reviews the heterogeneity in all domains of symptomatology and the failure to identify causes and effective treatments, and suggests that examining possible biology of specific, clearly defined symptoms will be more productive than trying to uncover the biology of a “syndrome” that does not really exist.

One would think that the sheer volume of research in the last 50 years would have settled the question of syndrome coherence, but since almost all studies with an autism group require both social communication deficits and repetitive and restricted behaviors, the existence of one without the other cannot be examined in these samples. Whether there is a strong link in the general population between the presence of these two general deficits (suggesting a continuum of an “autism trait”) or in their genetic liability has been argued positively (15) and negatively (16). Fein and Helt (17) argue that lack of co-occurrence or genetic linkage between the two autism domains in the *general population* does not bear directly on their relationship in a *neurodevelopmental syndrome*. This formidable problem echoes difficulties identified in other fields, such as the challenges of mapping from cognitive levels of analysis to neurobiology, to explain fundamental psychological processes such as language or vision (18). Developmental and clinical processes can help shed light on this mapping. Marr proposed that we gain explanatory power by describing problems or systems (or in the current case, syndromes) at three levels: the computational, the algorithmic, and the physical (19); addressing these levels will likely strengthen our theories of clinical phenomena.

An additional difficulty in considering the coherence of autism as a syndrome is the tremendous heterogeneity within each domain (11). Social impairment can range from aloofness and disinterest in other people, sometimes including parents, to a desire to socialize but with limited and inflexible social judgment. Language can range from complete lack of spoken language with very impaired language comprehension, to structural language that is within the normal range but affected by impaired social judgment. Intellectual ability can range from severe intellectual disability to superior cognitive functioning. The biological underpinnings of such a diverse set of abilities are also likely to be diverse.

### Prototypical autism

One approach to defining a more homogeneous syndrome rests with the idea of “prototypical” or “frank” autism, posited to be obvious to experienced clinicians within a few minutes (20, 21). Wieckowski et al. (21) found that clinicians were generally correct (high specificity) if they confidently detected autism in the first few minutes of observing very young children; their impressions of non-autism were less accurate (lower sensitivity). Mottron et al. (22, 23) suggest focusing on prototypical autism as a means of increasing the homogeneity of possible etiologies. Several issues limit the utility of this approach, including the difficulty of deciding who is expert enough to define prototypical autism, and the lack of success in aligning prototypical cases to underlying biology (17, 24).

### Non-biological causes of autism as a classifier

If “biological” refers to factors inherent to the development of the brain or other systems, usually genetic, then the overwhelming majority of autism cases are no doubt biological in origin. Although it may not be clear exactly where to draw the line, there is a fundamental distinction between causes inherent in the developing fetus vs. environmental causes (from intrauterine to early childhood environments), which might include toxic exposure, disease exposure, injury, preterm birth, or extreme deprivation. There is evidence that environmental deprivation can result in syndromes that would meet criteria for autism, as seen in children reared in neglectful institutions (25, 26) or those with severe congenital blindness (27). Although the relationship of blindness to autism remains controversial (28, 29), Jure et al. make a strong argument for blindness leading to true autistic behavior (27). Note that both situations involve sensory deprivation, which may impact early attachment and social communication (e.g., the development of infant-mother attachment). In cases where a non-biological factor is a prominent element, relatively good prognoses are likely if environmental change including sensory stimulation occurs early in development (28), validating severe environmental/sensory deprivation as a classifier.

### Individuals who lose the autism diagnosis (LAD)

Autism is characterized by distinct behavioral trajectories, demonstrated in longitudinal studies (30, 31). Uher and Rutter (32) suggest that developmental trajectory and outcome are relevant to reducing heterogeneity at the phenotypic level. One informative group is composed of individuals who amply met criteria for autism in earlier life and no longer do. Our published and ongoing studies of these individuals include only individuals with clear diagnoses of autism by the age of 5 years, who currently function within typical parameters, excluding borderline cases. We have described this group in detail (33), documenting their good social and adaptive skills (33, 34), typical academic abilities including reading comprehension (35), ability to focus on gestalt rather than overfocus on detail (36), and correct use of subtle dysfluency fillers (37). They received significantly more early behavioral intervention between ages 2 and 3 years than the still-autistic group (38), and were left with higher rates of ADHD than the control group (30). On observed and parent-reported measures of executive functioning, scores for the LAD group were within the average range, though scores on impulsivity, set-shifting, working memory, and planning were lower than scores in non-autistic controls (31). Other research groups have also reported on this subgroup whose symptoms remit with intervention (39–41).

In addition to advancing basic understanding of the biology of subgroups, treatment is another fundamental motivation for identifying autism syndromes and their underlying anatomy or physiology. By analogy, the best designs for bridges are informed by an understanding mechanical force. While it is possible to construct some bridges, such as timber-fall bridges, without this knowledge, bridges last longer, and withstand greater stress, when builders have explicit or implicit knowledge of these forces. Similarly, understanding the mechanisms that contribute to a syndrome at either the biological or behavioral level can be a potent contributor to effective treatment. For example, if individuals differ in patterns of activation in a reading task, one might hypothesize that those with prominent right hemisphere (likely compensatory) activation might benefit from approaches that incorporate strong visual and orthographic training components, while individuals with left hemisphere activation in areas similar to good readers, but with abnormalities (e.g., less connectivity, lower amplitude, slower response) might have phonological processing deficits that would benefit from intensive reading practice (42, 43).

### Learning about causal mechanisms from studying outcome status

Most autism studies, sensibly, include participants who meet diagnostic criteria for ASD; by definition, they have deficits in both DSM domains of (1) social communication and (2) the presence of repetitive and restricted behaviors (RRBs), making it nearly impossible to evaluate how these domains cluster. Another solution is to study individuals experiencing social disabilities, and then explore the presence, type, and extent of their RRBs, and also to do the opposite, taking a sample of individuals with significant RRBs and studying their social functioning. Such an enterprise could examine the interdependence of the two domains of impairment and the coherent syndrome status of autism. Certainly, looking at the emergence of earliest symptoms, and following how individual children respond to treatment—provides a critically important window into causal mechanisms. We further argue that understanding the forces that contribute to the steep developmental trajectory that characterizes LAD individuals provides a useful lens through which to conceptualize the mechanisms that underlie the symptoms of ASD. We can examine which specific features remit together in LAD, to understand more about which how symptoms of ASD “lawfully” co-occur and cluster; this approach offers a pathway to understanding the coherence of autism as a syndrome (13). Examining significant symptom remission in LAD, with a detailed and comprehensive evaluation of subclinical behaviors in both domains, and assessing brain anatomy and physiology, provide a pathway for understanding the coherence of ASD.

### Biology of LAD individuals

Is there any evidence that individuals whose autism symptoms remit have a distinctive biological underpinning of their autism, either anatomical or functional? One study examined head circumference growth in early childhood from medical records and found *no* differences between individuals whose autism later remitted and those who still met autism criteria, disappointing any hope of a straightforward anatomical marker (44). Following the example of examining brain activation in successfully remediated adult dyslexics (43), which found both increased activation in the usual reading areas plus compensatory activation in right hemisphere areas, we examined language-related brain activation in LAD, autistic, and non-autistic individuals. Eigsti et al. (45) found that LAD individuals showed a distinctive pattern of such brain activation, compared to autistic and non-autistic groups. Specifically, the LAD individuals had a small set of language-related activations similar to that found in the autistic individuals, and a large set of (likely compensatory) activations that was unique to LAD; there were *no* activation areas that were more like the typically developing controls than the autistic group. They concluded that, unlike the brain changes in improved dyslexia as reported by Eden et al. (41), LAD individuals showed some residual ASD patterns and extensive compensation, but little or no evidence of normalization of brain activations. Follow-up work indicated unique patterns of language-related neural specialization as it related to language abilities in these groups (46).

### Positive and negative aspects of LAD

While from one perspective, losing the diagnosis (and thus having fewer difficulties with social communication and fewer RRBs) is a positive outcome, it is not unambiguously so. Autistic self-advocates and others have raised concerns about one's identity as a member of the autism community and about the loss of the diagnosis which can be a helpful explanation of preferences (e.g., vocations that involve fewer social interactions with strangers) and abilities (e.g., efficient attention to detail). Prior changes in diagnostic entities, as in the removal of the Asperger's Disorder diagnosis in DSM-5 (47), led many to feel robbed of an important aspect of their identities. Additionally, “officially” losing the diagnosis may entail losing beneficial supports. More broadly, describing the loss of the diagnosis as a positive outcome implies that meeting criteria for autism is necessarily negative, a position vigorously rejected by many autism advocates (8). In response, our group has adopted the more neutral “loss of autism diagnosis, LAD” terminology (48).

## Conclusions

We have discussed the nature of syndromes, and whether it is possible to characterize autism in this way; approaches to defining autism, including prototypicality, non-biological causes of ASD and, especially, trajectories of change and outcomes (particularly focusing on LAD); the relevance of studying the neural circuitry associated with the steep developmental trajectories in LAD; and the pros and cons of losing the ASD diagnosis. Clearly, there has been slow and limited progress to date in understanding ASD via the medical model. Our group aims to better understand the causal mechanisms of ASD—(the underlying forces of tension, compression, and shear, in the bridge analogy)—by focusing on homogeneous groups that are subtyped by important clinical characteristics such as IQ, language level, and outcome status. While the strategy of studying smaller, more homogeneous subgroups in order to find links between phenotype and biology has not succeeded to date, this lack of success, which also characterizes research in schizophrenia, depression, and other conditions, reflects the enormity of the theoretical problem. In the long run, it seems to us that the slow but steady work of discovering and describing biological causes and then exploring the phenotypes associated with them is likely to yield the most solid long-range results. The parallel approach of defining more homogeneous subgroups, focusing on variables outlined here, offers the most effective path to specifying subgroups that will be useful in basic biological studies, and can help inform any needed treatment strategies.

## Author contributions

I-ME: conceptualization, data curation, funding acquisition, methodology, project administration, resources, and writing—original and editing. DF: conceptualization, funding acquisition, methodology, resources, and writing—original and editing. All authors contributed to the article and approved the submitted version.

## Funding

This research was funded by NIMH-1R01MH112687-01A1 to I-ME and DF (Co-PIs) and by NIDCD T32DC017703 to I-ME and E Myers.

## Conflict of interest

The authors declare that the research was conducted in the absence of any commercial or financial relationships that could be construed as a potential conflict of interest.

## Publisher's note

All claims expressed in this article are solely those of the authors and do not necessarily represent those of their affiliated organizations, or those of the publisher, the editors and the reviewers. Any product that may be evaluated in this article, or claim that may be made by its manufacturer, is not guaranteed or endorsed by the publisher.
